# Pregnancy, postpartum and breastfeeding: beliefs about women’s sexuality during this period

**DOI:** 10.1192/j.eurpsy.2024.562

**Published:** 2024-08-27

**Authors:** S. Bader, Z. Zran, M. Aloulou, A. Abdelmoula, A. Bouaziz, W. Abbes

**Affiliations:** ^1^Psychiatry; ^2^obstetric gynecology, University Hospital of Gabes, Gabes, Tunisia

## Abstract

**Introduction:**

Pregnancy,postpartum and breastfeeding is a very challenging period in the women’s life. Many shared false beliefs and perceptions about this period can influence a pregnant woman’s sexual life and couple.

**Objectives:**

to explore sexual satisfaction, misconceptions and beliefs about sexuality during pregnancy and postpartum in women.

**Methods:**

It was a cross-sectional study established over a period of 3 months from the June 1^st^, 2023 to August 31, 2023. This study focused on a population of pregnant postpartum and breastfeeding women recruited from outpatient consultations and inpatient of the obstetric gynecology department at the university hospital of Gabes, Tunisia. We used a pre-established sheet exploring socio-demographic data, medical and gyneco-obstetric history, informations concerning the marital relationship and the woman’s sexual activity and eight questions (yes or no / choosing an option) to explore the beliefs and perceptions about sexuality during pregnancy and postpartum. We administered the validated Arabic version of the Arizona Sexual Experiences Scale (ASEX) to assess sexual functioning.

**Results:**

Fifty-eight women were included. The average age was 35.6±5.5 years, they had a university level in 40% and they were unemployed in 74.2%. They were from an urban origin in 75%. They were pregnant in the first, second and third trimester in (15.6%, 15.6% and 25% respectively). They were in postpartum in 43.8% of cases with a cesarean delivery in 73.3% and breastfeeding in 56%. All women reported being on good terms with their spouses and satisfied with their sexuality. The usual frequency of sexual relations (SR) was (1/day: 22.6%, 1/week: 74.2%, 1/month: 3.2%). Only 3.4% masturbated and 5.17% had sexual fantasies. Among women, 55.1% believed that RS is not allowed in the first trimester, and 67.8% believed that it can harm the baby. Only 25% of women believed that RS is permitted throughout pregnancy. 58.1% believed that RS in the third trimester could induce early delivery, and 30% believed that it could harm the baby. They all believed that post-partum SR is only authorized after 40 days. Among the sample 22.6% believed that SR is not allowed during breastfeeding, and that it can harm the baby in 13% of cases. The mean ASEX score was 13 ± 4.3 and 47% had sexual dysfunction. Regarding the frenquency of SR, 25% reported wanting to reduce the frequency, 3.4% wanting to increase the frequency and 71.6% were neutral.

**Conclusions:**

A better understanding of the misconceptions and beliefs about sexuality during pregnancy and the post-partum period is needed to reduce restriction imposed on sexual activity during a normal pregnancy and to enhance marital harmony and the sexual life of the couple.

**Disclosure of Interest:**

None Declared

